# Supplementary material for: Long Non-Coding RNA ANRIL as a Potential Biomarker of Chemosensitivity and Clinical Outcomes in Osteosarcoma
Source: Int J Mol Sci. 2021 Oct 16;22(20):11168. doi: 10.3390/ijms222011168 (PMC8538287; doi:10.3390/ijms222011168)
Supplement: Supplementary file 1 [file ijms-22-11168-s001.zip › ijms-1381793-supplementary.pdf]

Supplemental Table S1: Characteristics of osteosarcoma cell lines utilized for in-vitro validation experiments

Cell Line	*Mutant Gene*	*Somatic Mutation Profile*	*Clinical Data Characteristics*
U-2 OS (ATCC® HTB-96™)	None	There are no coding mutations in CDKN2A, TP53, RB1, PTEN, and BRAF.	Caucasian, female, 15 y.o.
HOS (ATCC® CRL-1543™)	TP53; CDKN2A	CDKN2A homozygous c.1_471del471; TP53 homozygous c.467G>C p.R156P	Caucasian, female, 13 y.o.
Saos-2 (ATCC® HTB-85™)	RB1; TP53	RB1 homozygous c.2212_2787del576; TP53 homozygous c.1_1182del1182	Caucasian, female, 11 y.o.

Information was obtained from ATCC (https://www.atcc.org/)

Supplemental Figure S1: Increased *ANRIL* expression correlates with increased resistance to both cisplatin (A) and doxorubicin (B) in cancer cell lines. In-vitro drug response data were obtained from the Genomics of Drug Sensitivity in Cancer version 1 dataset (GDSC1; https://www.cancerrxgene.org/). *ANRIL* expression was imputed as previously described [24,25]. X-axis displays the log2 of the imputed *ANRIL* expression. Y-Axis displays the log of the reported therapeutic IC_{50} (μM) value.

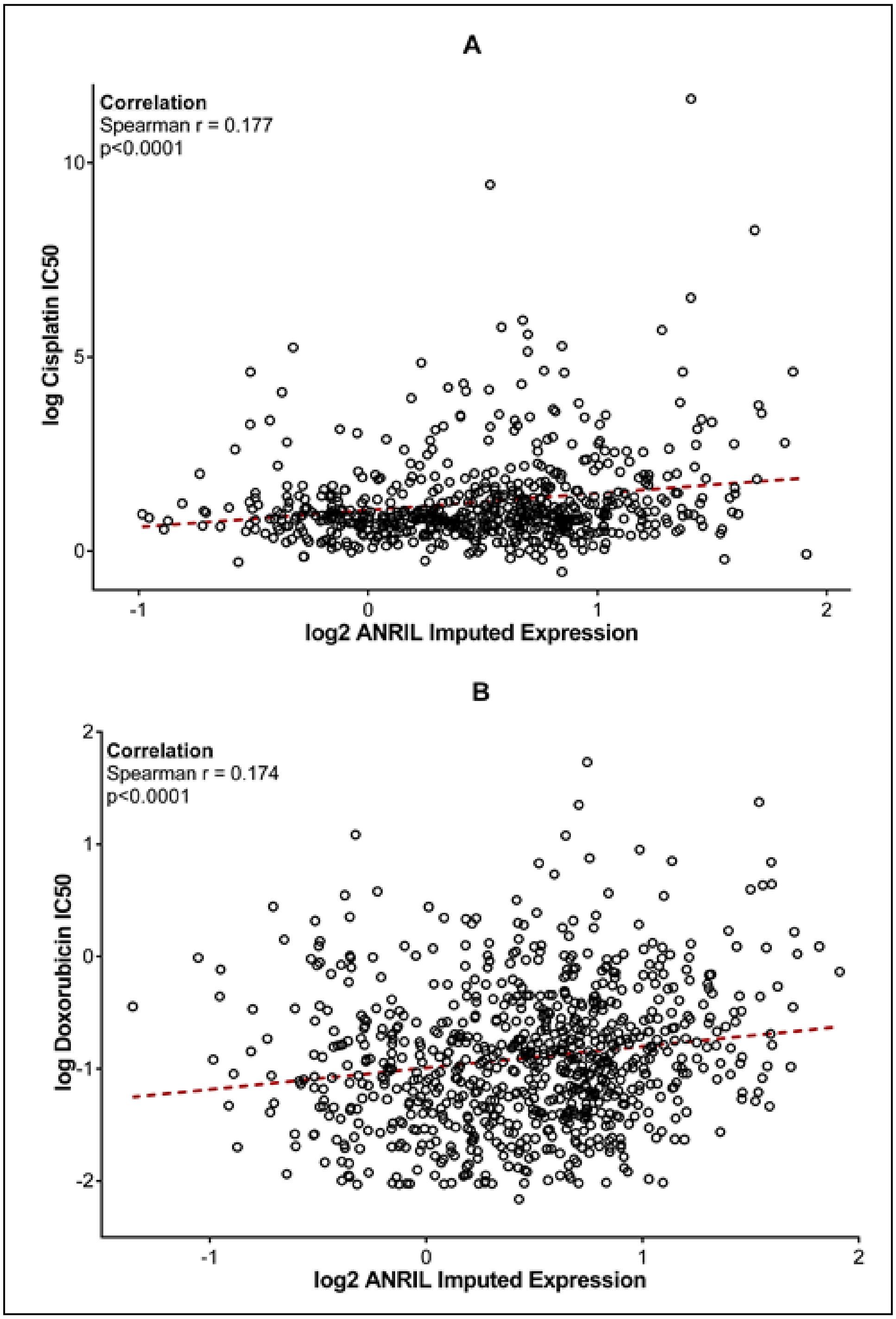

Supplemental Figure S2: lncRNA *ANRIL*, *CDKN2A*, *CDKN2B*, and *MTAP* gene expression in osteosarcoma cell lines HOS (dark grey), U2OS (light grey), and SAOS2 (red). Expression values for each gene are displayed as relative to the HOS cell line.

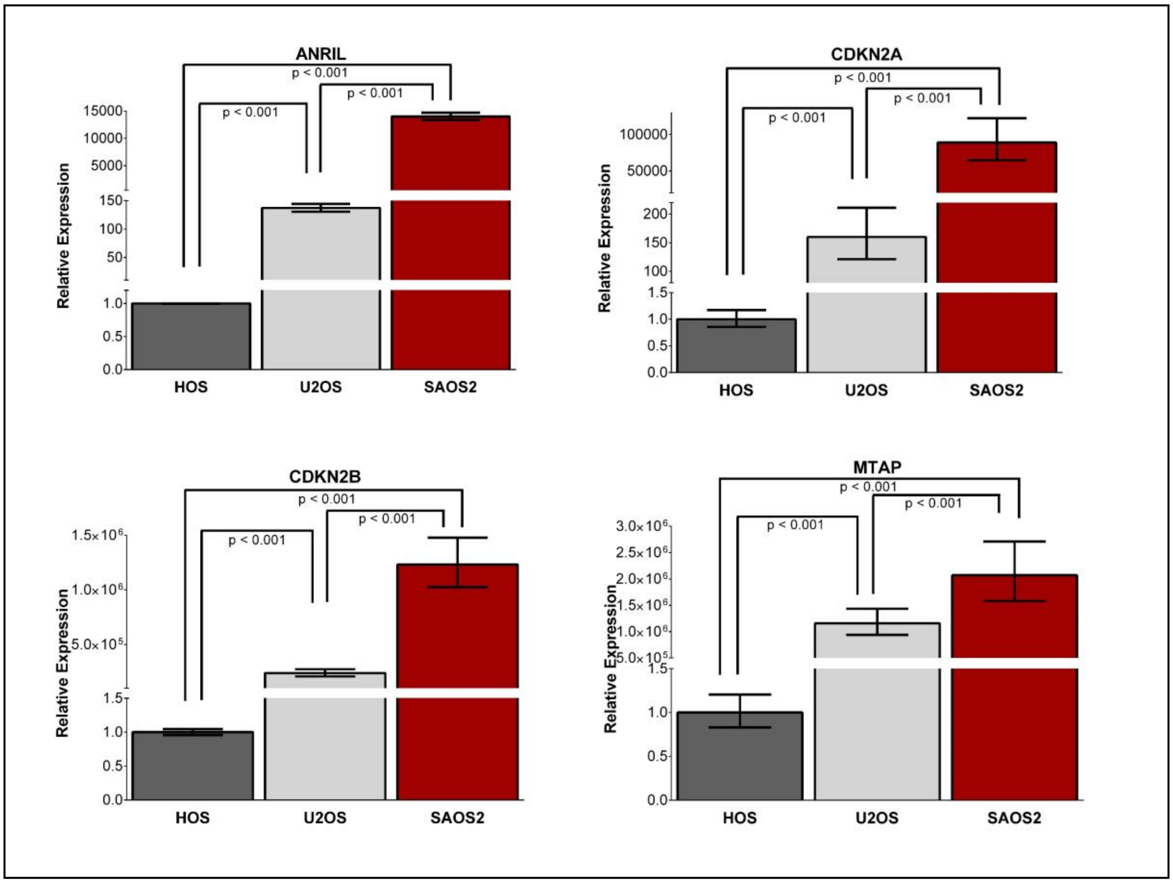

Supplemental Figure S3: Comparison of proliferation rates between ANRIL knockdown SAOS2 cells and control. Calculated measurements of proliferation were normalized to measurements taken at 24 hours after cell plating and siRNA transfection. *ANRIL* overexpression models are represented by lines and circles shaded in red. Empty vector expression control models are represented by lines and squares shaded in grey.

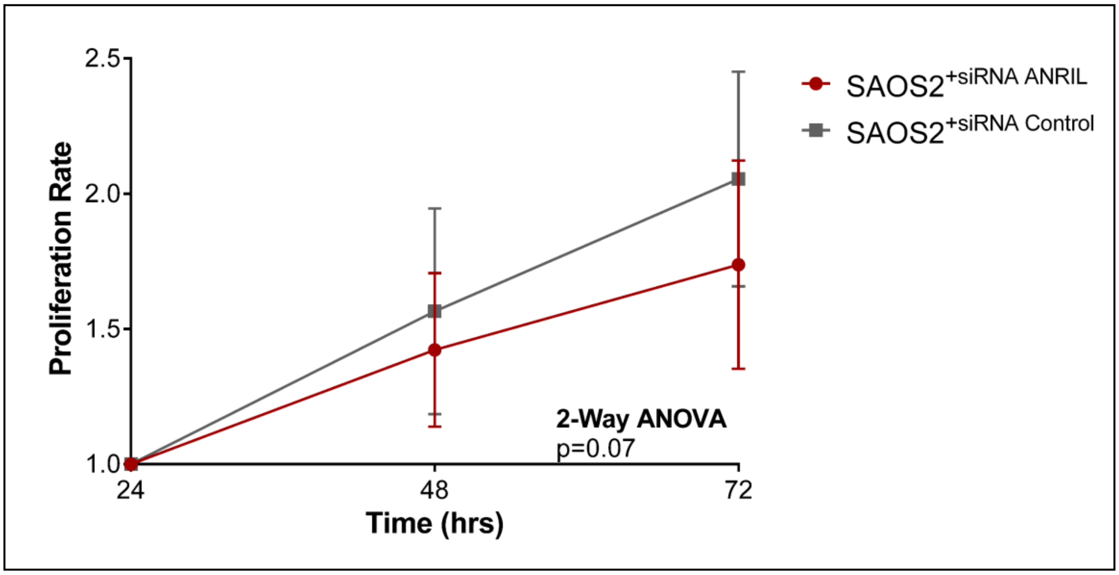

Supplemental Figure S4: Increased sensitivity measured at 24 and 48 hours post-treatment with cisplatin and doxorubicin following ANRIL knockdown in SAOS2 cells. Y-axis displays percent viability. X-axis displays log-transformed treatment concentrations. ANRIL knockdown SAOS2 cells are represented by lines and circles shaded in red. Control siRNA SAOS2 cells are represented by lines and squares shaded in grey. Each experimental condition was performed in triplicate and measurements were reported as the mean +/- standard deviation (S.D.) of three independent biological experiments. Dose points with minimal error (S.D. < 2) show no visible error bars.

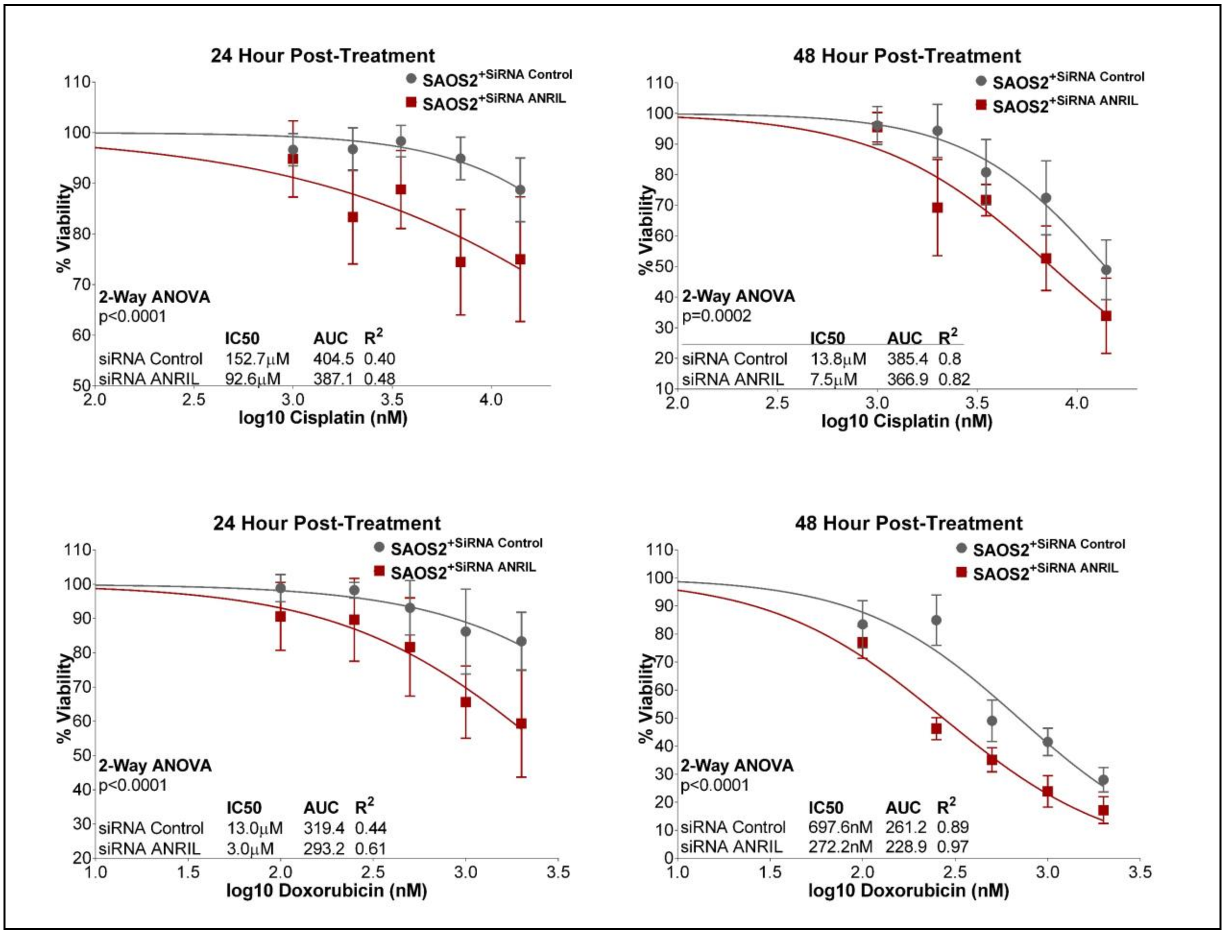

Supplemental Figure S5: Dose response curves of U2OS and HOS *ANRIL* overexpression models measured at 24 and 48 hours post-treatment with cisplatin and doxorubicin. Y-axis displays percent viability. X-axis displays log-transformed treatment concentrations. ANRIL overexpression models are represented by lines and circles shaded in red. Expression vector controls are represented by lines and squares shaded in grey. Each experimental condition was performed in triplicate and measurements were reported as the mean +/- standard deviation (S.D.) of three independent biological experiments. Dose points with minimal error (S.D. < 2) show no visible error bars.

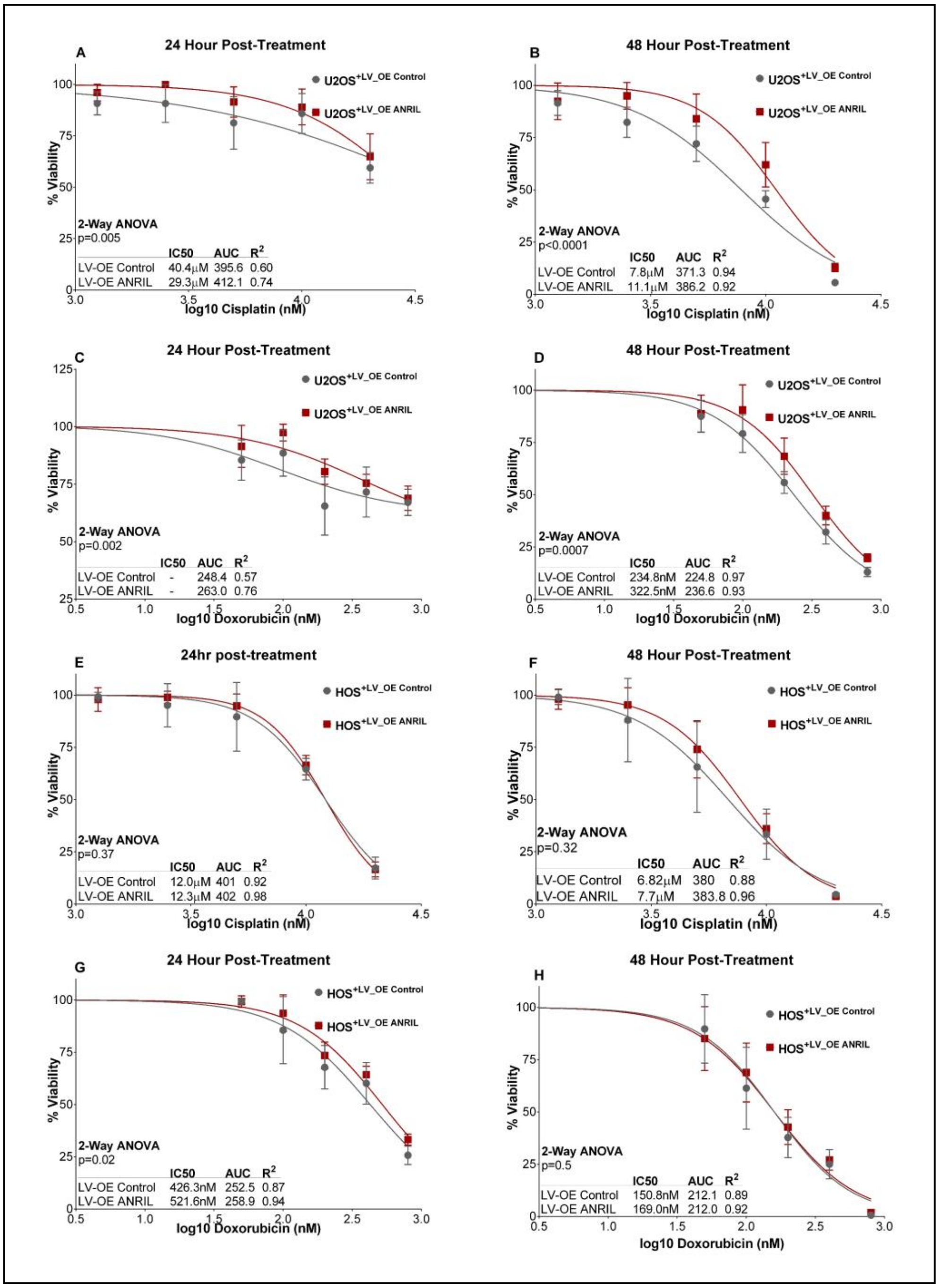

References:

24.Nath A, Geeleher P, Huang RS. Long non-coding RNA transcriptome of uncharacterized samplescan be accurately imputed using protein-coding genes. Brief Bioinform. 2020;21(2):637-648.

25.Nath A, Lau EYT, Lee AM, Geeleher P, Cho WCS, Huang RS. Discovering long noncoding RNApredictors of anticancer drug sensitivity beyond protein-coding genes. Proc Natl Acad Sci U S A. 2019;116:22020-22029.
